# Sleep EEG slow-wave activity in medicated and unmedicated children and adolescents with attention-deficit/hyperactivity disorder

**DOI:** 10.1038/s41398-019-0659-3

**Published:** 2019-11-28

**Authors:** Melanie Furrer, Valeria Jaramillo, Carina Volk, Maya Ringli, Robert Aellen, Flavia M. Wehrle, Fiona Pugin, Salome Kurth, Daniel Brandeis, Markus Schmid, Oskar G. Jenni, Reto Huber

**Affiliations:** 1 Child Development Center, University Children’s Hospital Zurich, 8032 Zurich, Switzerland; 2 Children’s Research Center, University Children’s Hospital Zurich, 8032 Zurich, Switzerland; 3 Pulmonary Clinic, University Hospital Zurich, 8091 Zurich, Switzerland; 4 Department of Child and Adolescent Psychiatry and Psychotherapy, Psychiatric Hospital, University of Zurich, 8032 Zurich, Switzerland; 5 Department of Child and Adolescent Psychiatry and Psychotherapy, Central Institute of Mental Health, Medical Faculty Mannheim, Heidelberg University, 68159 Mannheim, Germany; 6 Center for Integrative Human Physiology, University of Zurich, 8057 Zurich, Switzerland

**Keywords:** Neuroscience, Biomarkers, Physiology, ADHD

## Abstract

Slow waves (1–4.5 Hz) are the most characteristic oscillations of deep non-rapid eye movement sleep. The EEG power in this frequency range (slow-wave activity, SWA) parallels changes in cortical connectivity (i.e., synaptic density) during development. In patients with attention-deficit/hyperactivity disorder (ADHD), prefrontal cortical development was shown to be delayed and global gray matter volumes to be smaller compared to healthy controls. Using data of all-night recordings assessed with high-density sleep EEG of 50 children and adolescents with ADHD (mean age: 12.2 years, range: 8–16 years, 13 female) and 86 age- and sex-matched healthy controls (mean age: 12.2 years, range: 8–16 years, 23 female), we investigated if ADHD patients differ in the level of SWA. Furthermore, we examined the effect of stimulant medication. ADHD patients showed a reduction in SWA across the whole brain (−20.5%) compared to healthy controls. A subgroup analysis revealed that this decrease was not significant in patients who were taking stimulant medication on a regular basis at the time of their participation in the study. Assuming that SWA directly reflects synaptic density, the present findings are in line with previous data of neuroimaging studies showing smaller gray matter volumes in ADHD patients and its normalization with stimulant medication.

## Introduction

Despite attention-deficit/hyperactivity disorder (ADHD) is one of the most studied developmental disorders from child- to adulthood, the genetic and neuronal factors underlying the development of ADHD are not fully understood^1^. Yet, a wide range of neuroimaging studies revealed that lower gray matter volumes in different brain regions, e.g. the basal ganglia^2,3^, and lower cortical thickness^4,5^ are associated with the disorder. According to longitudinal magnetic resonance imaging (MRI) data, there is evidence that a delay in brain maturation underlies these differences^5–8^.

In recent years, a new imaging modality proved useful in tracking brain maturation in humans, i.e. high-density electroencephalography (hd EEG) recordings during sleep^9^. Using this technique, studying sleep EEG slow waves, oscillations of about 1–4.5 Hz characterizing deep non-rapid eye movement (NREM) sleep, offer a promising readout of the functional output of neuronal connectivity^10,11^. Slow-wave activity (SWA) is defined as the EEG power in the slow wave frequency band. During the first two decades of life, SWA follows an inverted U-shaped curve^10,12^, similar to gray matter development. Moreover, the predominant location of SWA undergoes a posterior–anterior shift across the scalp from early childhood to late adolescence^13^, which again parallels cortical maturation^14,15^. In children with ADHD, SWA was shown to be relatively increased over central brain regions^16^. Since this pattern resembles that of healthy children of younger age, it supports the assumption that ADHD is characterized by a delay in cortical maturation. Apart from this study, little is known about ADHD-related topographical alterations in SWA during sleep.

In the present study, we included data of all-night recordings assessed with high spatial resolution sleep hd EEG of 50 children and adolescents with ADHD and 86 age- and sex- matched healthy controls. Our aim was to investigate whether ADHD patients differ from healthy controls in terms of sleep SWA across the cortex. Additionally, we aimed to assess the effects of psychostimulants on SWA, because standard prescribed drugs, i.e. psychostimulants, not only improve specific and broader clinical symptoms, but also seem to normalize gray matter maturation^3,17,18^.

## Methods

### Participants

Fifty patients (13 female) between 8 and 16 years (mean: 12.2 years) diagnosed with ADHD (33 combined type, 14 predominantly inattentive type, 3 unknown) were included in the analysis (Table 1). Fifteen were recruited from the Department for Child and Adolescent Psychiatry at the University of Zurich (14 diagnosed according to DSM-IV criteria and 1 according to DSM-V criteria) and 27 from a private children’s practice in Zurich Oerlikon (all diagnosed according to DSM-IV criteria). For 8 participants the information about what diagnostic criteria were applied was not available. Diagnoses were not verified. All subjects participated in one of three studies performed between 2010–2012 (data of 9 participants published in ref. ^16^), 2013 (unpublished) and 2017 (unpublished), respectively. 10 ADHD patients (10–16 years, mean: 12.8 years, 2 female) reported regular intake of stimulant medication for the treatment of ADHD (methylphenidate, 18–80 mg/day, mean: 42 mg/day) including the morning of measurement. Another 18 patients (8–16 years, mean: 12.2 years, 5 female) reported regular intake of psychostimulants but refrained from medication during the 24 h before the evening of the night measurement. Sixteen of them received methylphenidate (Concerta®, Ritalin®, Ritalin LA®, Medikinet®) with dosages ranging from 10 to 46 mg per day (mean: 28 mg/day) and two lisdexamphetamine (Elvanse®, both 30 mg/day). According to personal reports, medication was usually taken five (workdays) to seven days per week. However, we did not use a standardized drug diary in the weeks before the experimental night. Five patients were medicated with methylphenidate during the time of measurement, but details on the concentration and last time of drug intake before the sleep assessment were not assessed. Six patients (9–14 years, mean: 12.7 years, 1 female) received psychostimulant medication in the past but had stopped medical treatment before the start of the study (from several weeks up to 2 years) and did no longer receive stimulant drugs, and 11 were drug-naïve (9–15 years, mean: 11.6 years, 2 female). Details of diagnosis across these medication subgroups can be found in Table 1.

**Table 1:** Diagnosis of ADHD patients across medication subgroups.

	Med in past *n* = 6	Unmed *n* = 11	Med day before *n* = 18	Med measurement day *n* = 10	Med unknown details
Center diagnosed (CAP/CP/unknown)	1/4/1	8/3/0	1/16/1	5/4/1	0/0/5
Diagnose (DSM-IV/DSM-V/unknown)	5/0/1	10/1/0	17/0/1	9/0/1	0/0/5
Type (combined/inattentive/unknown)	2/4/0	9/1/1	13/5/0	7/2/1	2/2/1

Patients were diagnosed by the Department for Child and Adolescent Psychiatry at the University of Zurich (CAP) or by a private children’s practice in Zurich Oerlikon (CP) according to DSM-IV or DSM-V criteria. The diagnostic criteria from eight participants were unknown. The resulting diagnosis was either combined type or predominantly inattentive type. Numbers indicate the number of patients belonging to a certain category. Five medicated ADHD patients were not assigned to a medication subgroup, because details on the concentration and last time of drug intake before the sleep assessment were not available (Med unknown details).

Eighty-six sex- and age-matched healthy control participants (mean age: 12.2 years, range: 8–16 years, 23 female) who were never diagnosed with a psychiatric disorder were included in the analysis. Matching of the control group was done as follows: all children in the same age range as the ADHD patients (8–16 years) were selected from three studies that were performed in 2008–2013^13,19,20^ and from a study conducted in 2017 (unpublished), resulting in 121 possible healthy control participants. From this pool, the maximal amount of participants was chosen such that their age (mean and standard deviation) as well as the proportion of sexes precisely matched the ADHD group, leading to a sample size of *n* = 86. Recruitment of control participants was done via local schools, advertisements and social media.

Of note: both, in the patient and control group, subjects were pooled from several, independent studies. All studies were performed in the same rooms, with the same equipment (EEG-system, electrodes etc.), followed the same general procedure and the experimenters were always master- or PhD students of the same research group. Furthermore, none of the participants reported to suffer from a diagnosed sleep disorder at the time of measurement.

To evaluate overall cognitive differences between the groups, the intelligence quotient (IQ) was estimated from either German (short-) versions of the WISC-III or WISC-IV intelligence test^21,22^ (59 controls, 27 ADHD patients) or from the TONI-4^23^ (16 controls). No differences in IQ were found between the control group and any of the ADHD subgroups. Within the ADHD subgroups, IQ was significantly lower in patients who were medicated in the past as compared to unmedicated patients (*p* < 0.01, ANOVA with Tukey-Kramer post hoc test, Supplementary Table S1). However, because of missing data in 23 ADHD and 11 control participants, statistical power is limited. In terms of sex, proportions were similar across medication subgroups and the control group (ADHD groups: 17, 18, 28, and 20%; control group: 27% females).

For all participants, written informed consent was obtained from a parent and/or legal guardian and in addition from adolescent participants 14 years and older. All studies were approved by the local ethics committee (Kantonale Ethikkommission Zürich, Switzerland) and performed according to the Declaration of Helsinki.

### Procedure

During 7 days prior to the sleep assessment, participants were instructed to stick to a regular sleep–wake rhythm with deviations of bed and get up times of less than 1 h. Regular bedtimes were verified with wrist accelerometry (Actiwatch Plus, AW4, Cambridge Neurotechnology or GeneActiv accelerometer, ActivInsight Ltd.) and sleep diaries including bed and get up times and alcohol/caffeine consumption. Participants were further asked to restrict their caffeine consumption to a maximum amount of two servings per day. Medication was only permitted in patients with ADHD. Whole-night EEG recordings took place in the sleep laboratory of the University Children’s Hospital Zurich. Sleep times were scheduled according to habitual bedtimes.

### Recording and preprocessing of EEG data

All-night sleep hd EEG was recorded (Electrical Geodesics Sensor Net for long-term monitoring, 128 channels, Electrical Geodesics Inc., EGI, Eugene, OR, USA). All electrodes were filled with an electrolyte gel (electro-gel, Electro-Cap International). Four additional gold electrodes (Grass Technologies, West Warwick, RI, USA) were attached to the chin (EMG) and earlobes. EEG data were referenced to the vertex and sampled at 500 Hz (filtered between 0.01 and 200 Hz). Data were offline bandpass filtered between 0.5 and 40 Hz and down-sampled to 128 Hz. Sleep was visually scored in 20 s epochs according to the AASM Manual for scoring sleep^24^. Artifacts were rejected based on visual inspection during scoring on a 20 s basis. Additionally, artifacts were removed on a semiautomatic basis if power exceeded a threshold based on mean power values in the 0.75–4.5 Hz and 20–30 Hz bands. On average, 17% of epochs were excluded in the ADHD group and 14% in the control group. Data of each participant were then re-referenced to the average value across all electrode channels excluding the lowest row.

### Data analysis

Sleep parameters and data of sleep diaries were compared between the groups using ANCOVA with cofactor age and Tukey-Kramer post hoc tests. EEG power was calculated for all channels and for 20 s epochs by means of a fast Fourier transform routine (Hanning window, averages of five 4 s epochs, frequency resolution of 0.25 Hz). EEG channels with poor signals were excluded and the power value was interpolated using spherical linear interpolation. The lowest row of electrodes was excluded for analysis resulting in 109 included channels. In contrast to our previous study^16^, the larger sample size allowed the comparison of absolute SWA values (without any normalization). Therefore, power values were averaged over the first hour of artifact-free NREM sleep (stages N2 and N3) and 1–4.5 Hz and then logarithmized to ensure normal distribution of the data (Shapiro-Wilk tests). Electrode-wise group comparisons were performed using two-tailed Student’s unpaired *t* tests and statistical nonparametric mapping (SnPM), using a supratreshold cluster analysis^25^ to control for multiple comparisons. The subgroup analysis was performed by means of both electrode-wise and across electrodes ANCOVA with cofactor age. For pairwise comparisons, Tukey-Kramer post hoc tests were used because of unequal sample sizes, but equal within-group variances across the groups (*p* = 0.91, Barlett test). The alpha level of all statistical tests was set to 0.05. All analyses were performed with the software package MATLAB (MathWorks, R2014a/R2017b) and R i386 (version 3.4.2).

## Results

### Sleep diary

We compared sleep–wake history and caffeine consumption according to sleep diaries between the following groups: ADHD patients who had received stimulant medication in the past but not at the time of their participation in the study (“ADHD-med in past”, *n* = 6), ADHD patients who had never received stimulant medication (“ADHD-unmed”, *n* = 11), ADHD patients who received psychostimulants on a regular basis but refrained from taking them during the 24 h prior to the EEG assessment (“ADHD-med day before”, *n* = 18), ADHD patients who received psychostimulants on a regular basis including the morning of measurement (“ADHD-med measurement day”, *n* = 10) and healthy controls (*n* = 86). Bed time, get up time and time in bed during the week before the measurement did not differ between groups. Furthermore, no differences were observed in mean caffeine consumption per day, which was very low for all groups (Supplementary Table S1). Because the level of SWA is dependent on the immediate sleep–wake history^26^, we calculated time spent awake and time spent in bed during the last ~24 h before the measurement. These parameters were not different between the groups (Supplementary Table S1).

### Sleep parameters

For a comparison of sleep architecture and quality of the recording night, sleep parameters were calculated from visually scored sleep. EEG recordings were significantly longer in the ADHD group “med-day before” as compared to the control group, leading to significantly more time spent in bed (31.27 min; *p* = 0.02) and a longer sleep duration (48.45 min, *p* *<* 0.01). Thus, we expressed all sleep stages and wake after sleep onset as percentage of total sleep time or time spent in bed. We found no group differences neither in the composition of sleep stages, nor in sleep latency or wake after sleep onset, with the exception of that the ADHD group “med-day before” spent significantly less time in stage N1 as compared to the control group (*p* < 0.05) and that the ADHD group “med-day of measurement” had a longer sleep latency than the groups “ADHD-unmed” (*p* < 0.01), “ADHD-med day before” (*p* < 0.01) and healthy controls (*p* < 0.01). Sleep quality as represented by sleep efficiency was high in all groups (Supplementary Table S2).

### Difference in SWA between ADHD patients and healthy controls

We focused our analyses of SWA on the first hour of artifact-free NREM sleep (stages N2 and N3) for the following reasons: (1) for some participants EEG data were only available for the beginning of the night due to EEG-net removal, (2) the EEG recordings were shorter in healthy controls, and (3) most consolidated sleep results in a most stable estimate of sleep pressure at the beginning of the night. To assure that the composition of the first NREM sleep hour is similar between groups, we compared absolute length, sleep architecture as well as time awake during this time window and found no differences between ADHD and healthy control participants (Supplementary Table S2).

**Fig. 1:**
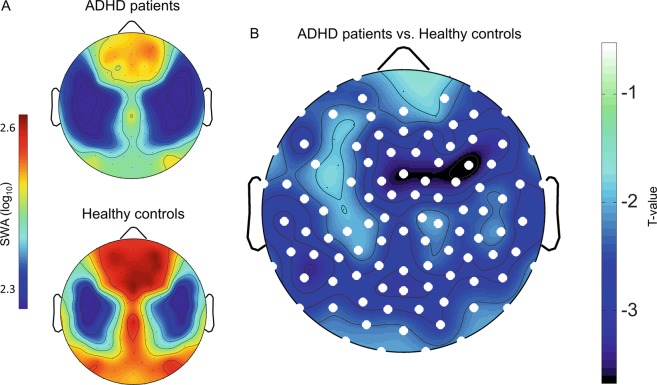
Comparison of slow-wave activity between ADHD patients and healthy controls. **a** Topographical representation of SWA (log_10_ EEG power 1–4.5 Hz) within the first hour of artifact-free NREM sleep (N2 and N3, average over participants) for ADHD patients (*n* = 50) and healthy controls (*n* = 86). Values are color-coded and scaled to maximum (red) and minimum (blue) of the control group. **b** Distribution of t-values over the scalp resulting from electrode-wise Student’s unpaired *t* test comparing the ADHD’s with the control group’s log_10_ SWA. Significant electrodes (*p* < 0.05, unpaired Student’s *t* test) are indicated as white dots. SWA was significantly reduced in ADHD patients in 101 electrodes (−20.5%, *t* = −2.8, *p* < 0.01).

To investigate local SWA changes, electrode-wise comparisons of SWA (log_10_) between ADHD and healthy control participants were performed. SWA of ADHD patients showed a significant decrease of 20.5 ± 0.32% (mean over electrodes ± SEM) in 101 out of 109 electrodes compared to healthy controls (*p* < 0.01, two-sided Student’s unpaired *t* test, Fig. 1).

### Effect of medication on SWA

**Fig. 2:**
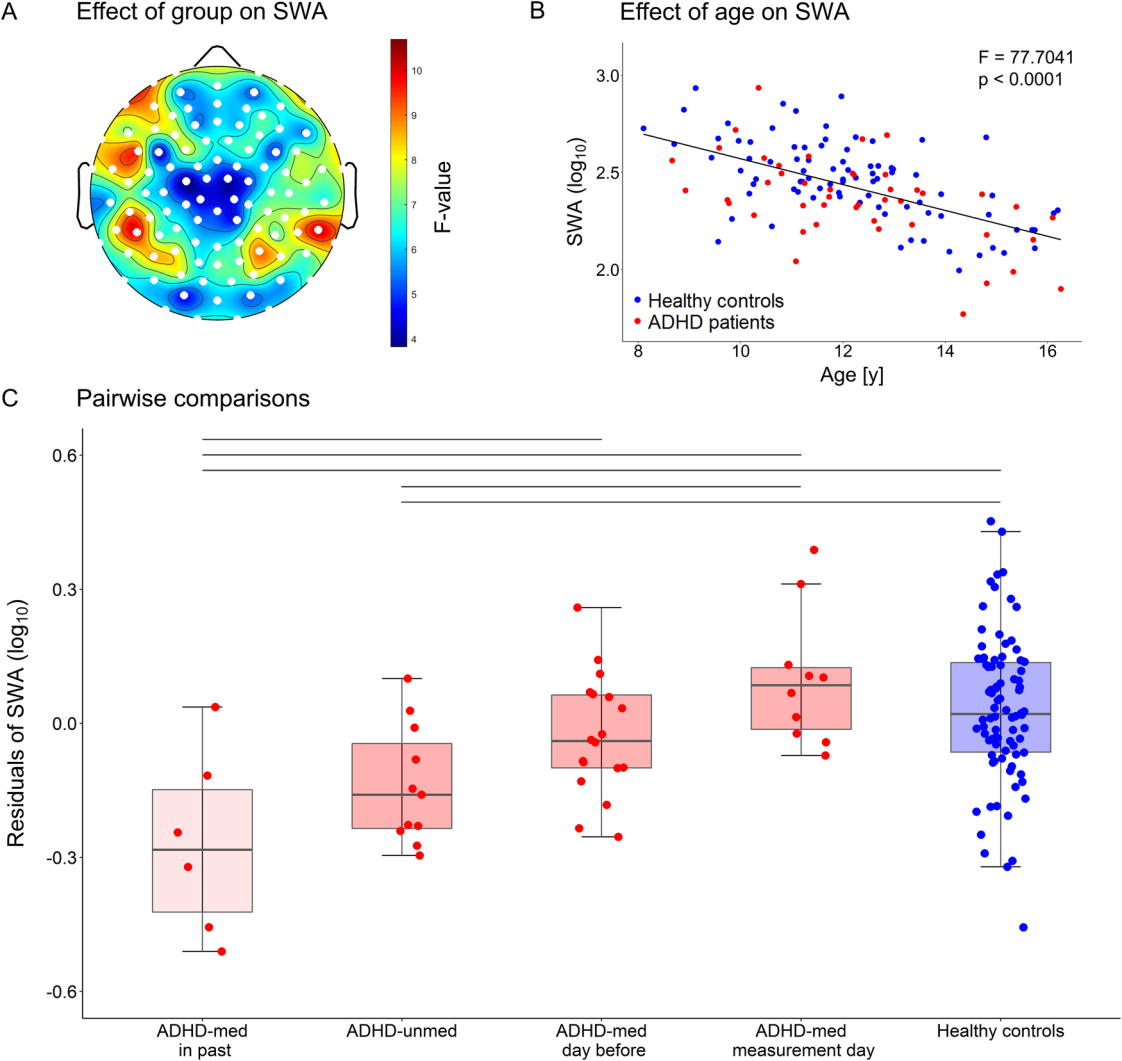
ANCOVA testing the effect of group and age on slow-wave activity and pairwise comparisons of groups. **a** Electrode-wise F-values resulting from ANCOVA (effect of group on log_10_ SWA after controlling for age) illustrated on the scalp-model. Groups are: ADHD patients who received stimulant medication in the past but not during the time of the measurement (“ADHD-med in past”, *n* = 6), ADHD patients who never received stimulant medication (“ADHD-unmed”, *n* = 11), ADHD patients who received psychostimulants on a regular basis but refrained from taking them during the last 24 h before the evening of the measurement (“ADHD-med day before”, *n* = 18), ADHD patients who received psychostimulants on a regular basis including the morning of the measurement (“ADHD-med measurement day”, *n* = 10) and healthy controls (*n* = 86). Significant electrodes (*p* < 0.05) are indicated as white dots. The effect of group on SWA was significant across all electrodes (*F* = 7.98, *p* < 0.01). **b** Averaged log_10_ SWA over all electrodes plotted against age. ANCOVA revealed a significant effect of age on SWA (*F* = 77.7, *p* < 0.01), and no interaction effect (*F* = 0.06, *p* = 0.99). **c** Pairwise comparisons of groups (Tukey-Kramer post hoc tests). Boxplots show the residuals of log_10_ SWA with the lower and upper hinges corresponding to the first and third quartiles. The whiskers extend from the hinge to the largest/smallest value at most 1.5 times the inter-quartile range from the hinge. Lines above boxplots indicate significant pairs (*p* < 0.05). SWA was significantly lower in both the “ADHD-med in past” and the “ADHD-unmed” group compared to both the “ADHD-med measurement day” and the control group. Furthermore, SWA was lower in the “ADHD-med in past” compared to the “ADHD-med day before” group. Results of the “ADHD-med in past” group have to be taken with caution, since their sleep during the investigated time window seemed to be more disturbed.

To identify possible medication effects on the observed decrease in SWA, we performed a subgroup analysis with the medication groups specified above. Because subgroups do not perfectly match in terms of age, which is known to be negatively correlated with SWA, we included age as a cofactor in the following analyses. Electrode-wise analysis of covariance (ANCOVA) with cofactor age revealed a significant effect of group on SWA in each electrode (Fig. 2a). Due to this consistent difference across the scalp, we averaged SWA across electrodes for the following analyses. When performing the same ANCOVA with these average values, as expected, the overall group effect was highly significant (*F* = 7.98, *p* < 0.01). Moreover, a strong effect of age on SWA was observed (*F* = 77.7, *p* < 0.01, Fig. 2b), but there was no significant interaction between group and age (*F* = 0.06, *p* = 0.99). Pairwise comparisons (Tukey-Kramer post hoc tests) showed that SWA was decreased in both the “ADHD-unmed” group and the “ADHD-med in past” group as compared to both the “ADHD-med measurement day” group and the control group. In addition, the “ADHD-med in past” group showed significantly lower SWA than the “ADHD-med day before” group (Fig. 2c). Since the level of SWA might also be influenced by disturbed sleep during the investigated time window, we performed additional ANCOVAs with cofactor age to test the effect of group on the composition of the first NREM sleep hour. We found a significant effect of group on the proportion of N1 and wake (*p* < 0.05). Pairwise comparisons revealed that the “ADHD-med in past” group experienced significantly more wake and N1 as compared to the “ADHD-med day before” group (Supplementary Table S2). The result of significantly lower SWA in the “ADHD-med in past” group has thus to be interpreted with caution, since it might result from more fragmented sleep.

## Discussion

The main result of this study is that sleep EEG SWA is reduced across the scalp in children and adolescents with ADHD compared to healthy controls. Interestingly, we found that the SWA reduction was mostly driven by patients without stimulant medication at the time of their participation in the study. Both of these findings are in agreement with the well-established anatomical differences in ADHD patients. Specifically, several studies found that children with ADHD exhibit widespread reductions in gray matter volume and cortical thickness^3,4,27,28^. Moreover, psychostimulant medication has been shown to normalize both gray matter^3,18^ and cortical thickness^29^. This parallelism between sleep SWA and cortical gray matter might not be surprising given that several studies have established a direct relationship between the two^10,30^.

Our knowledge about how slow waves are generated supports a close relationship between SWA and gray matter. Slow waves on the cortical surface reflect slow oscillations of cortical neurons^31^. These slow oscillations of cortical neurons during deep sleep are the result of an unique alternating activity pattern between active “on” states and completely silent “off” states. The more neurons are involved and the more synchronized they display these “on”/“off” states, the larger the amplitude of the surface slow wave^32^. Thus, higher synaptic connectivity and stronger synaptic connections result in larger and highly synchronized networks of neurons involved in slow oscillations and in more SWA recorded on the cortical surface.

This relationship might explain why particularly during development, when major reorganization processes take place, sleep SWA parallels major maturational changes in cortical gray matter, i.e. (1) the inverted U-shape time course during the first two decades of life^10,12,14,33^, (2) the regional maturation along the posterior-anterior axis^13,15,34,35^, and (3) experience-dependent changes in synaptic plasticity, which are favored by processes of brain maturation^36^. These close relationships seem not to be exclusive for gray matter, but specific aspects of slow waves may also relate to the maturation of white matter microstructure^37^.

Sleep EEG SWA represents a promising readout of age-dependent changes in functional neuronal connectivity and may thus complement the anatomical markers of brain maturation^36^. As such, this electrophysiological readout potentially contributes to the understanding of why ADHD patients show reduced cortical gray matter for the following reasons. It was proposed that a delay in cortical maturation underlies the brain anatomical differences^7^. Indeed, in our previous work published in 2013 by Ringli and coworkers, we concluded, based on the topographical differences in SWA, that children with ADHD show a less mature pattern than healthy control participants. Our novel analysis with a larger study population, allowing a comparison of absolute SWA, reveals a global reduction of SWA in patients with ADHD. This observation does not exclude that additional local changes are present. The directionality of the relationship between sleep SWA and a maturational delay is unknown. In other words, whether decreased SWA supports a cortical developmental delay, or alternatively whether a maturational delay results in decreased SWA remains to be established with longitudinal studies. Two observations, which both support an active role of SWA in cortical gray matter maturation, encourage the exploration in more detail: (1) cortical maturation assessed by SWA precedes gray matter maturation measured by means of MRI^38^ and (2) good evidence exists that SWA plays a critical role in synaptic plasticity^39^. Thus, reduced SWA in ADHD patients may actively contribute to the delay of brain development.

Finally, we would like to discuss major confounders and potential limitations of the study.

First, the attenuated effect on SWA in the medicated group may be caused by a withdrawal effect rather than by medication itself, because participants were asked to refrain from taking medication on the day of EEG measurement. This, however, is unlikely because patients who refrained from taking their medication on the day of measurement did not significantly differ from patients who continued taking psychostimulants on the day of measurement. On the other hand, psychostimulants may have an acute effect on SWA, since only patients who continued taking their medication on the day of EEG assessment, but not those who withdrew from it, differed significantly from unmedicated patients. Additionally, in agreement with previous studies^40,41^, we observed an acute medication effect on sleep onset latency (Supplementary Table S2), supposedly due to the arousal-enhancing effects of psychostimulants. Thus, based on our data we cannot conclude if the main effect of stimulant medication is mediated by a long-term or an immediate effect.

Second, symptom severity could mediate some of the group differences we observe. Unfortunately, across the extended time window in which measurements were taking place, no standard procedures for the assessment of symptom severity was conducted. Consistent across all patients is that they had a diagnosed ADHD and fulfilled the same exclusion and inclusion criteria. However, our data do not allow to explore the relationship between present findings and symptom severity. Furthermore, the proportion of children recruited by one or the other institution varies across medication subgroups. While the majority of unmedicated patients was recruited by one institution, most medicated patients who refrained from medication intake on the day of measurement were recruited by the other (Table 1).

Third, the level of SWA depends on experiences and activities performed during the day^36,42,43^. Therefore, it is possible that alterations in SWA are not only a result of different brain anatomy between children with and without ADHD, but also of differing daytime activities. To account for differences in time awake prior to the EEG assessment and potential effects on SWA^26^, we incorporated the evaluation of sleep diaries, which showed that sleep–wake history did not differ between ADHD and control participants. It is thus very unlikely that the decrease in SWA was caused by lower sleep pressure in ADHD patients.

Fourth, one might speculate that global SWA changes in ADHD patients result from disturbed sleep in this patient group. By looking at the architecture of the first NREM sleep hour we could show that durations of wake and sleep stage N1 did not differ between ADHD and control subjects. A more direct measure for sleep fragmentation is the number of awakenings. Thus, we compared awakening counts during the first NREM sleep hour of ADHD patients (*n* = 50) with those of healthy controls (*n* = 86) and found that ADHD patients tended to wake up even fewer times than healthy controls (*p* = 0.04). When performing a medication-subgroup analysis, we counted significantly more awakenings for the ADHD group “med in past” as compared to the ADHD group “med day before” (*p* = 0.04, Tukey-Kramer post hoc test), which is consistent with different durations of wake and N1 in the same subgroups (see above). Thus, apart from the “ADHD-med in past” group, decreased SWA in ADHD patients cannot be explained by more sleep fragmentation.

Fifth, undetected sleep problems in the ADHD group might have influenced our results. Sleep disorders, such as obstructive sleep apnea and periodic limb movements, were shown to affect a high percentage of ADHD patients^44^. In the present study, we excluded patients who reported to suffer from sleep problems and/or a diagnosed sleep disorder, but did not perform a polysomnography allowing a clinical assessment of the participant’s sleep. It is, however, unlikely that the ADHD group differed from the control group in terms of sleep problems, since we did not detect differences in the amount of awakenings, the length of wake after sleep onset, nor in sleep–wake history.

Finally, also power in other frequency ranges may show differences between ADHD and control participants. Relatedly, recent studies also reported lower EEG power in the sigma-frequency range (12–16 Hz) in children with ADHD^45,46^, however, without focusing on topographical differences. We limited our hypothesis driven approach to the SWA frequency range to avoid a reduction in statistical power because of multiple comparisons.

Having these limitations in mind, our results show that children and adolescents diagnosed with ADHD experience a reduction in EEG SWA during NREM sleep. Stimulant medication seems to have normalizing effects on SWA. These findings are in line with neuroimaging studies showing decreased levels of gray matter, possibly caused by a maturational delay in ADHD patients. This parallelism between ADHD-related gray matter changes and SWA changes needs to be further investigated by assessing both MRI and sleep EEG in a large patient sample.

## Supplementary information

Supplemental Material
