# Supplementary material for: Sleep EEG slow-wave activity in medicated and unmedicated children and adolescents with attention-deficit/hyperactivity disorder
Source: Transl Psychiatry. 2019 Nov 28;9:324. doi: 10.1038/s41398-019-0659-3 (PMC6883036; doi:10.1038/s41398-019-0659-3)
Supplement: Supplementary file 1 — Supplemental Material [file 41398_2019_659_MOESM1_ESM.docx]

Supplemental Material for

Sleep EEG slow-wave activity in medicated and unmedicated children and adolescents with attention-deficit/hyperactivity disorder

Melanie Furrer^1,2^, Valeria Jaramillo^1,2^, Carina Volk^1,2^, Maya Ringli^1^, Robert Aellen^1^, Flavia M. Wehrle^1,2^, Fiona Pugin^1^, Salome Kurth^3^, Daniel Brandeis^4,5,6^, Markus Schmid^1^, Oskar G. Jenni^1,2,6^, Reto Huber^1,2,4^

1. Child Development Center, University Children’s Hospital Zurich, 8032 Zurich, Switzerland
2. Children’s Research Center, University Children’s Hospital Zurich, 8032 Zurich, Switzerland
3. Pulmonary Clinic, University Hospital Zurich, 8091 Zurich, Switzerland
4. Department of Child and Adolescent Psychiatry and Psychotherapy, Psychiatric Hospital, University of Zurich, 8032 Zurich, Switzerland
5. Department of Child and Adolescent Psychiatry and Psychotherapy, Central Institute of Mental Health, Medical Faculty Mannheim, Heidelberg University, 68159 Mannheim, Germany
6. Center for Integrative Human Physiology, University of Zurich, 8057 Zurich, Switzerland

**Supplementary Table S1**

**Table S1. Sleep diary.** Sleep diaries including bed and get up times as well as daily caffeine consumption were completed during 7 days prior to the sleep assessment. Reported values are the means (mean) and standard deviations (SD) of 6.17 ± 0.16 (mean ± SEM) nights reported by the ADHD patients and of 6.24 ± 0.10 nights reported by healthy controls. Time spent in bed represents the time between bed and get up time. For the day before the assessment, time awake was calculated by subtracting the get up time of the morning before the measurement from the time of lights off (reported by the examiner) at the measurement. P-values result from ANCOVA testing. Overall, these variables indicate that sleep-wake history and caffeine consumption prior to the EEG assessment did not differ between groups. Age did not differ between the medication subgroups and healthy controls (ANOVA). Estimated IQ (WISC-III, WISC-IV, short version of WISC-IV, TONI-4) differed between the ADHD subgroups “med in past” and “unmed” (ANOVA, Tukey-Kramer post-hoc test).

|  | **(1) ADHD-med in past** | | | **(2) ADHD-unmed** | | | **(3) ADHD-med day before** | | | **(4) ADHD-med measurement day** | | | **(5) Healthy controls** | | | **p-value** | **Pairwise comparisons** |
| --- | --- | --- | --- | --- | --- | --- | --- | --- | --- | --- | --- | --- | --- | --- | --- | --- | --- |
|  | n | mean | SD | n | mean | SD | n | mean | SD | n | mean | SD | n | mean | SD |  |  |
| **Questionnaires and Age** |  |  |  |  |  |  |  |  |  |  |  |  |  |  |  |  |  |
| Age [y] | 6 | 12.70 | 1.94 | 11 | 11.60 | 1.64 | 18 | 12.17 | 2.13 | 10 | 12.84 | 1.95 | 86 | 12.16 | 1.84 | 0.6002 |  |
| estimated IQ | 4 | 95.95 | 10.33 | 8 | 124.60 | 11.71 | 6 | 118.04 | 6.45 | 4 | 107.75 | 10.09 | 75 | 114.10 | 13.37 | **0.0071** | 1≠2 |
|  |  |  |  |  |  |  |  |  |  |  |  |  |  |  |  |  |  |
| **Sleep diary (week before measurement)** | |  |  |  |  |  |  |  |  |  |  |  |  |  |  |  |  |
| Bed time | 5 | 22.01 | 1.07 | 10 | 21.47 | 0.51 | 17 | 21.91 | 0.84 | 10 | 21.86 | 0.96 | 81 | 21.91 | 0.72 | 0.5462 |  |
| Get up time | 5 | 7.12 | 0.31 | 10 | 7.24 | 0.47 | 17 | 7.42 | 0.63 | 10 | 7.62 | 1.23 | 81 | 7.32 | 0.70 | 0.6476 |  |
| Time spent in bed [min] | 5 | 9.11 | 1.10 | 10 | 9.76 | 0.75 | 17 | 9.51 | 0.89 | 10 | 9.75 | 0.89 | 81 | 9.45 | 0.81 | 0.2718 |  |
| Caffeine consumption [mg/day] | 5 | 9.62 | 6.61 | 10 | 17.46 | 21.71 | 18 | 7.85 | 4.83 | 10 | 7.04 | 10.02 | 83 | 9.57 | 19.04 | 0.4238 |  |
|  |  |  |  |  |  |  |  |  |  |  |  |  |  |  |  |  |  |
| **Sleep diary (day and night before measurement)** | | |  |  |  |  |  |  |  |  |  |  |  |  |  |  |  |
| Time spent in bed [min] | 5 | 8.47 | 1.48 | 10 | 9.53 | 0.92 | 17 | 9.79 | 1.33 | 10 | 9.48 | 0.9882 | 81 | 9.47 | 0.97 | 0.1605 |  |
| Time awake [min] | 5 | 15.04 | 0.81 | 10 | 14.83 | 1.10 | 17 | 14.49 | 1.18 | 10 | 14.57 | 0.6649 | 79 | 14.58 | 1.09 | 0.7925 |  |
| Get up time | 5 | 6.80 | 0.54 | 10 | 7.05 | 1.37 | 17 | 7.48 | 1.00 | 10 | 7.02 | 0.8379 | 81 | 7.38 | 0.94 | 0.4363 |  |
| Lights off = Start EEG-recording | 6 | 21.93 | 0.56 | 11 | 21.78 | 0.65 | 18 | 21.97 | 0.58 | 10 | 21.58 | 0.3304 | 83 | 21.93 | 0.58 | 0.2129 |  |

**Supplementary Table S2**

**Table S2. Sleep architecture**. Sleep parameters were calculated from visual scoring of sleep stages. Sleep efficiency was calculated by dividing total sleep time by total time in bed. Wake after sleep onset is expressed as the percentage of total time in bed. The different sleep stages (NREM sleep, stage N1, stage N2, stage N3 and rapid eye movement sleep) are expressed as percentage of total sleep time. Three ADHD patients were excluded, because the recording did not cover the whole night. P-values result from ANCOVA testing. For pairwise comparisons, Tukey-Kramer post-hoc tests were applied. Sleep architecture differed between the groups in terms of total time in bed, total sleep time, sleep latency and stage N1. Architecture of the first NREM sleep hour was calculated for the time window containing the first 60 min of artefact-free N2 and N3 sleep. The ADHD group “med in past” was significantly more awake and in stage N1 as compared tho the “med day before” group during this time window.

|  | **(1) ADHD-med in past** | | | **(2) ADHD-unmed** | | | **(3) ADHD-med day before** | | | **(4) ADHD-med measurement day** | | | **(5) Healthy controls** | | | **p-value** | **Pairwise comparisons** |
| --- | --- | --- | --- | --- | --- | --- | --- | --- | --- | --- | --- | --- | --- | --- | --- | --- | --- |
|  | n | mean | SD | n | mean | SD | n | mean | SD | n | mean | SD | n | mean | SD |  |  |
| **Sleep architecture** |  |  |  |  |  |  |  |  |  |  |  |  |  |  |  |  |  |
| Total time in bed [min] | 5 | 540.73 | 19.13 | 11 | 549.61 | 37.40 | 17 | 556.75 | 41.72 | 9 | 559.56 | 17.17 | 86 | 525.48 | 42.64 | **0.0030** | 3≠5 |
| Total sleep time [min] | 5 | 473.20 | 45.40 | 11 | 495.82 | 45.76 | 17 | 513.45 | 50.97 | 9 | 463.59 | 99.71 | 86 | 465.00 | 47.20 | **0.0087** | 3≠5 |
| Sleep efficiency [%] | 5 | 87.51 | 7.64 | 11 | 90.17 | 4.85 | 17 | 92.18 | 5.32 | 9 | 82.69 | 17.27 | 86 | 88.63 | 7.25 | **0.0463** | n.s. |
| Sleep latency [min] | 5 | 22.80 | 10.64 | 11 | 19.39 | 13.08 | 17 | 16.92 | 12.10 | 9 | 48.19 | 38.62 | 86 | 20.11 | 11.94 | **0.0000** | 2≠4; 3≠4; 4≠5 |
| Wake after sleep onset [%] | 5 | 8.74 | 7.45 | 11 | 6.62 | 4.22 | 17 | 5.23 | 4.78 | 9 | 5.83 | 3.91 | 86 | 8.03 | 6.40 | 0.3858 |  |
| NREM sleep [%] | 5 | 81.22 | 3.91 | 11 | 77.44 | 3.91 | 17 | 77.34 | 4.15 | 9 | 80.30 | 4.86 | 86 | 79.26 | 4.62 | 0.2342 |  |
| Stage N1 [%] | 5 | 5.33 | 2.51 | 11 | 5.78 | 2.64 | 17 | 4.83 | 1.86 | 9 | 5.05 | 1.45 | 86 | 7.46 | 3.95 | **0.0211** | 3≠5 |
| Stage N2 [%] | 5 | 44.78 | 7.07 | 11 | 48.03 | 4.69 | 17 | 46.71 | 5.95 | 9 | 46.79 | 13.68 | 86 | 47.54 | 6.75 | 0.8494 |  |
| Stage N3 [%] | 5 | 31.10 | 8.13 | 11 | 23.63 | 5.93 | 17 | 25.80 | 6.96 | 9 | 28.46 | 15.43 | 86 | 24.26 | 8.87 | 0.2959 |  |
| REM sleep [%] | 5 | 18.78 | 3.91 | 11 | 22.56 | 3.91 | 17 | 22.66 | 4.15 | 9 | 19.70 | 4.86 | 86 | 20.74 | 4.62 | 0.2342 |  |
|  |  |  |  |  |  |  |  |  |  |  |  |  |  |  |  |  |  |
| **Composition of first NREM sleep hour** |  |  |  |  |  |  |  |  |  |  |  |  |  |  |  |  |  |
| Absolute length [min] | 6 | 104 | 49.73 | 11 | 88.76 | 52.05 | 18 | 73.50 | 5.91 | 10 | 77.90 | 19.37 | 86 | 77.69 | 21.83 | 0.0901 |  |
| Wake [%] | 6 | 15.91 | 20.76 | 11 | 2.06 | 2.85 | 18 | 0.15 | 0.28 | 10 | 5.52 | 15.07 | 86 | 2.58 | 8.84 | **0.0093** | 1≠3 |
| Stage N1 [%] | 6 | 4.92 | 3.86 | 11 | 1.46 | 2.57 | 18 | 0.70 | 1.01 | 10 | 0.99 | 1.15 | 86 | 2.38 | 3.04 | **0.0108** | 1≠3 |
| Stage N2 [%] | 6 | 34.82 | 20.18 | 11 | 30.40 | 12.40 | 18 | 28.22 | 12.11 | 10 | 26.08 | 15.98 | 86 | 31.94 | 14.90 | 0.5503 |  |
| Stage N3 [%] | 6 | 44.35 | 34.45 | 11 | 63.60 | 18.54 | 18 | 68.98 | 13.17 | 10 | 65.62 | 20.98 | 86 | 61.94 | 18.51 | 0.0957 |  |
| REM sleep [%] | 6 | 0.00 | 0.00 | 11 | 2.48 | 5.93 | 18 | 1.95 | 4.09 | 10 | 1.78 | 4.78 | 86 | 1.16 | 3.26 | 0.6218 |  |
